# Complete atrioventricular block with prolonged asystolic pause at loop recorder monitoring in a young patient with brugada syndrome and conduction abnormalities: cause of syncope or incidental finding?

**DOI:** 10.1093/ehjcr/ytad367

**Published:** 2023-08-02

**Authors:** Nicolò Martini, Manuel De Lazzari, Federico Migliore

**Affiliations:** Department of Cardio-Thoraco-Vascular Sciences and Public Health, University of Padua, Via Giustiniani 2, Padova, 35128, Italy; Department of Cardio-Thoraco-Vascular Sciences and Public Health, University of Padua, Via Giustiniani 2, Padova, 35128, Italy; Department of Cardio-Thoraco-Vascular Sciences and Public Health, University of Padua, Via Giustiniani 2, Padova, 35128, Italy

## Case report

**Figure 1:**
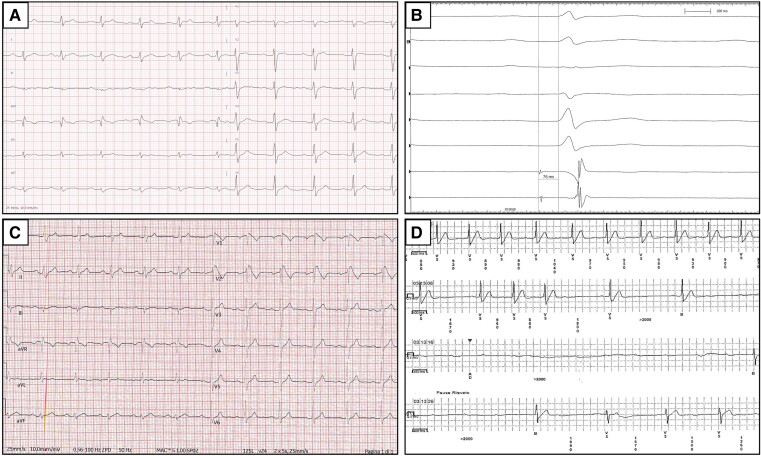
Panel *A*. 12-lead electrocardiogram (ECG) showing sinus rhythm, first-degree atrioventricular block, and non-specific intraventricular conduction delay. Panel *B*. HV interval at the electrophysiological study showing a prolongation of the HV interval. Panel *C*. 12-lead ECG during fever showing a spontaneous type 1 Brugada ECG pattern. Panel *D*. Implantable loop recorder monitoring showing a nocturnal episode of complete atrioventricular block with a prolonged asystolic pause (>15 s).

A 32-year-old healthy man with unremarkable familial and past medical history presented to the emergency department for remittent fever. Laboratory exams showed increased liver enzymes while abdomen ultrasound revealed hepatic enlargement and hyperechogenicity. He was diagnosed with acute hepatitis and successfully treated with antipyretics and antibiotics. First electrocardiogram during apyrexia showed first-degree atrioventricular block (AVB) and non-specific intraventricular conduction delay (*Figure 1*, *Panel A*). In the following days, he underwent two sudden and diurnal syncopal episodes without prodromes, the first while he was walking and the latter at rest. The 12-lead electrocardiogram (ECG) during fever revealed a type 1 Brugada ECG pattern (*Figure 1*, *Panel C*). Echocardiogram and cardiac magnetic resonance were unremarkable. Telemetry monitoring showed nocturnal episodes of marked sinus bradycardia. The electrophysiological study was negative for the induction of ventricular arrhythmias, but a prolonged HV interval was recorded (76 milliseconds) (*Figure 1*, *Panel B*).^1^ At the genetic testing, no pathogenetic or likely pathogenetic variants emerged. A Shanghai score of 5 was calculated, thus he was diagnosed with a ‘high risk’ Brugada Syndrome (BrS). An implantable cardioverter defibrillator implantation (ICD) was proposed, but he refused. Thus, he underwent an implantable loop recorder (ILR) implantation. After 3 months, at remote monitoring, a nocturnal episode of complete AVB with an asystolic pause of 15 s was recorded (*Figure 1*, *Panel D*).^1^ The electrocardiogram showed a spontaneous type 1 Brugada ECG pattern with high-risk features as first-degree AVB and prominent S wave in lead I.^1^ ICD implantation was performed with uneventful follow-up. Paroxysmal complete AVB may be a rare arrhythmic presentation in BrS. We have described a unique case of a life-threatening complete AVB in a young BrS patient with syncope and conduction abnormalities. ILR may have a potential role in the diagnosis, management, and follow-up of these events. The association between syncopal episodes and the arrhythmic event remains uncertain.

## Data Availability

The data underlying this article will be shared on reasonable request to the corresponding author.

## Notes

**Consent**: The authors confirm that written consent for submission and publication of this case report including images and associated text has been obtained from the patient in line with COPE guidance.

**Funding**: None declared.
